# Regional Variability in COVID-19 Case Fatality Rate in Canada, February–December 2020

**DOI:** 10.3390/ijerph18041839

**Published:** 2021-02-14

**Authors:** Eunha Shim

**Affiliations:** Department of Mathematics, Soongsil University, Seoul 06978, Korea; alicia@ssu.ac.kr

**Keywords:** COVID-19, case fatality rate, Canada, mortality, public health

## Abstract

A total of 475,214 COVID-19 cases, including 13,659 deaths, had been recorded in Canada as of 15 December 2020. The daily reports of confirmed cases and deaths in Canada prior to 15 December 2020 were obtained from publicly available sources and used to examine regional variations in case fatality rate (CFR). Based on a factor of underestimation and the duration of time from symptom onset to death, the time-delay adjusted CFR for COVID-19 was estimated in the four most affected provinces (Quebec, Ontario, Alberta, and British Columbia) and nationwide. The model-based adjusted CFR was higher than the crude CFR throughout the pandemic, primarily owing to the incorporation in our estimation of the delay between case reports and deaths. The adjusted CFR in Canada was estimated to be 3.36% nationwide. At the provincial level, the adjusted CFR was the highest in Quebec (5.13%)—where the proportion of deaths among older individuals was also the highest among the four provinces—followed by Ontario (3.17%), British Columbia (1.97%), and Alberta (1.13%). Provincial-level variations in CFR were considerable, suggesting that public health interventions focused on densely populated areas and elderly individuals can ameliorate the mortality burden of the COVID-19 pandemic.

## 1. Introduction

Since severe acute respiratory syndrome coronavirus-2 (SARS-CoV-2) was identified in China in December 2019, more than 70 million cases of coronavirus disease 2019 (COVID-19) have been reported globally by the World Health Organization, with 1,599,922 related deaths as of 15 December 2020 [1]. In Canada, the federal government established its Emergency Operations Centre on 15 January 15 2020, and the first case of the virus was confirmed on 27 January 2020, when an individual who had returned to Toronto from Wuhan, Hubei, China tested positive [2]. On 27 February, the Federal/Provincial/Territorial Public Health Response Plan for Biological Events was activated, and it was at level 3–“escalated,” which triggered response planning [3]. Until March, all cases were linked to recent travel to an endemic country; however, on 5 March, Canada’s first known case of community transmission was reported in British Columbia (B.C.). On 7 March, Canada’s first long-term care (LTC) facility outbreak was recorded in B.C., followed by Canada’s first death related to COVID-19, confirmed on 9 March in B.C. [4] As cases of community transmission were confirmed, the government of Ontario was the first to announce that public schools would be closed from 14 March to 5 April 2020 [5]. Public health emergencies were announced in Quebec on 13 March, followed by Ontario and Alberta on 17 March, and British Columbia on 18 March. As a result, by mid-March, all of Canada’s provinces and territories had declared states of emergency.

Following the announcement of public health emergencies, provinces and territories implemented school closures, restrictions on entry, closures of non-essential businesses, prohibitions on gatherings, and mandatory self-isolation for travelers. On 21 March, Canada closed its border with the United States, with some exceptions for freight movement and essential workers [6]. In addition, the Canadian government restricted border access in general, barring entry to almost all travelers from all countries [6]. By mid-to-late summer, it appeared as though Canada had flattened the curve of the pandemic, with a steady decline in active cases. However, near the end of the summer, a resurgence of cases was observed in most provinces and territories. Across all provinces, Canada recorded a sudden spike in the number of COVID-19 cases, from approximately 300 per day in August to a record high of over 6000 in December. The daily number of new cases was over 3.5 times more than it was at its peak in April, with deaths averaging approximately 120 per day. On 23 September, it was declared that Canada was experiencing a second wave of the virus. As a result, new restrictions and response measures from provincial governments were put in place, including new regional lockdowns.

As of 15 December 2020, 475,214 cases of COVID-19 had been reported in Canada, including 385,975 recoveries and 13,659 deaths. Although confirmed cases had been reported in all of Canada’s provinces and territories, with Nunavut being the last to report its first confirmed case on 6 November, most of these cases have been in Canada’s two most populous provinces, Ontario and Quebec. The Canadian government anticipates 11,000–22,000 deaths over the course of the pandemic, assuming intensive epidemic control [2]. In Canada, outbreaks of COVID-19 in LTC facilities have contributed substantially to the mortality associated with COVID-19 [7]. Syed et al. reported that 81% of COVID-19-related deaths in Canada have occurred in LTC facilities—almost twice the average rate of countries within the Organization for Economic Cooperation and Development (OECD) [8]. Residents in LTCs are mostly elderly, and thus, are more likely to have comorbidities than the average Canadian. In addition, it was found that nursing home residents are five times more likely to die of COVID-19 compared with community-dwelling older adults [9]. Moreover, it was estimated that nursing home residents accounted for 35% of COVID-19 deaths in the United States and 66%–81% of deaths in Canada [10]. In Ontario, for instance, COVID-19 has resulted in over 1400 deaths of residents and caregivers in LTC facilities [11]. In addition, the risk of transmission of COVID-19 within and among LTC facilities is much higher than that among the general population, as has also been observed in outbreaks of other respiratory infections [12]. As a result, these factors amount to a relatively high case incidence and case fatality rate (CFR) among LTC facility residents compared to the general population, which has had a large influence on the average CFR observed in Canada.

The CFR is defined as the proportion of people who die from a disease among all those diagnosed over a certain period of time. Thus, the CFR is an important measure for judging the extent and severity of diseases such as COVID-19, allowing public health officials to set priorities in targeted interventions to reduce risk severity. Estimates of the crude CFR for COVID-19 show considerable variations among different countries, in different regions of the same country, and at different stages of the outbreak. This is partially owing to differences in health control policies, healthcare availability, medical standards, and detection efficiency. Initial studies have reported an estimated 3% for the global CFR of COVID-19 [13], whereas country-specific crude CFRs range from the lowest rates in Germany (0.7%) and South Korea (2.4%), to the highest rates in Canada (4.9%), the United States (5.4%), Spain (6.0%), the Netherlands (7.4%), and Italy (9.3%) [14,15,16].

Traditionally, the CFR is calculated by dividing the number of deaths attributable to a disease by the total number of diagnosed individuals. However, this method is known to result in biased CFR estimates, caused by the delay between disease onset and knowledge of the final outcome [17]. In this study, given the need for timely CFR estimates when making public health decisions, we provide real-time estimates of the CFR during the COVID-19 pandemic in Canada—nationwide and in its four most affected provinces—through 15 December 2020, while adjusting for the delay between disease onset and death. These estimates were then used to assess provincial-level variations in the pandemic’s severity in Canada.

## 2. Methods

In this study, the virulence of COVID-19 was assessed by measuring the risk of death, expressed as CFR. The daily number of confirmed cases and deaths associated with COVID-19 in Canada from 31 January to 15 December 2020 was obtained from publicly available sources [18,19,20,21,22]. These data were categorized by geographic area, including Canada (national) and the four individual provinces where 95% of the total deaths associated with COVID-19 nationwide have occurred: Quebec, Ontario, Alberta, and British Columbia.

The CFR is interpreted as the conditional probability of death caused by infection from the virus. However, the denominator of the CFR formula also includes infected people who have not yet died from the disease, but will do so in the future. Thus, the delay between infection and death might result in bias when calculating the CFR. In statistics, the need to account for the amount of time required for cases to die is referred to as right censoring [23]. Specifically, during the course of updating epidemiological observations, the case count data is right censored.

To minimize this bias, the following equation was used:

(1) $$p_{t}=\frac{b_{t}}{u_{t}}$$

where $p_{t}$ is the unbiased estimator of the CFR, $b_{t}$ is a crude biased estimated CFR calculated at time *t*, and $u_{t}$ is the factor of underestimation [24]. To calculate the factor of underestimation $u_{t}$, incidence data and the distribution of time from disease onset to death were used. Specifically, it was assumed that the factor of underestimation, $u_{t}$, was given by the distribution of the time from onset to death, f(s), where f(s) was the density of a gamma distribution with mean T and coefficient of variation v [24]. Here, a mean (T) of 13.59 days and a standard deviation ($v^{2}$) of 7.85 days were assumed [15,25]. To account for the uncertainty in time from disease onset to death, the distribution f(s) was varied in the CFR estimates by sampling the mean from a normal distribution with a mean of 13.59 days and a standard deviation of one day. With this standard deviation, the mean survival interval from disease onset to death varied between 2 and 5.5 weeks, consistent with recent findings [26,27].

Based on this assumption, we calculated the factor of underestimation,

(2) $$u_{t}={\left(1+rTv^{2}\right)}^{-1/v^{2}},$$

where r is the growth rate. The growth rates of the epidemic during COVID-19 outbreaks at the provincial and national levels were calculated based on the maximum likelihood method. The moment-generating function was then used to determine the underestimation factor for the adjusted CFR on each calendar day by running Monte Carlo simulations with 1000 independent replications [24].

## 3. Results

As of 15 December 2020, a total of 475,214 cases of COVID-19 had been reported in Canada, including 13,659 deaths. British Columbia was initially considered most at risk because of its interconnectivity with Asia; however, the province reported 43,463 cases (869 per 100,000, or 9% of COVID-19 cases in Canada) and 668 deaths. Ontario and Quebec, Canada’s two most populous provinces, reported the majority of the infections (66%), with 144,396 (1006 per 100,000) and 167,276 (1994 per 100,000) cases, respectively (Table 1). Alberta reported 83,327 cases (1935 per 100,000). Confirmed cases have been reported in all of Canada’s provinces and territories.

**Figure 1:**
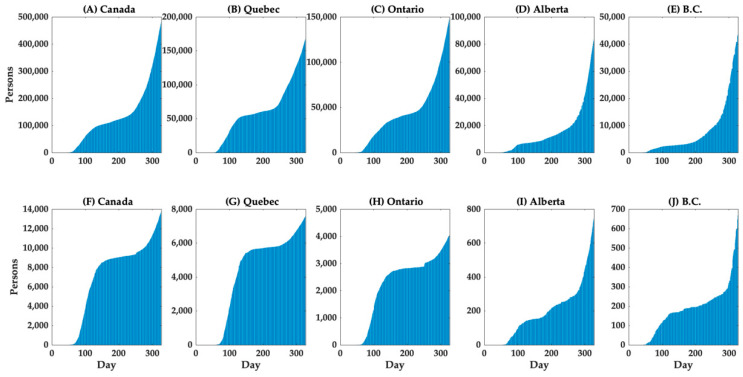
Temporal distribution of cases (top row) and deaths by area (bottom row), 1 February–15 December 2020. Cumulative cases in (**A**) Canada (total), (**B**) Quebec, (**C**) Ontario, (**D**) Alberta, and (**E**) British Columbia (B.C.), and cumulative deaths in (**F**) Canada (total), (**G**) Quebec, (**H**) Ontario, (**I**) Alberta, and (**J**) British Columbia (B.C.) are shown.

The cumulative cases and deaths in Canada (nationwide), Quebec, Ontario, Alberta, and British Columbia are shown in Figure 1. The curve of the cumulative number of deaths grows after the cumulative number of cases grows, although there was a more rapid increase in the number of deaths in Quebec. Furthermore, the associated mortality burden appears to be much higher in Quebec than in other provinces; specifically, among the 13,659 COVID-19-associated deaths in Canada, 55.4% were in Quebec and 29.2% were in Ontario, while 5.4% and 4.9% were in Alberta and British Columbia, respectively (Table 1). Notably, the fatality risk increased dramatically with age, with the crude CFR among individuals aged 80 and above reaching 25.71% (Table 2).

**Figure 2:**
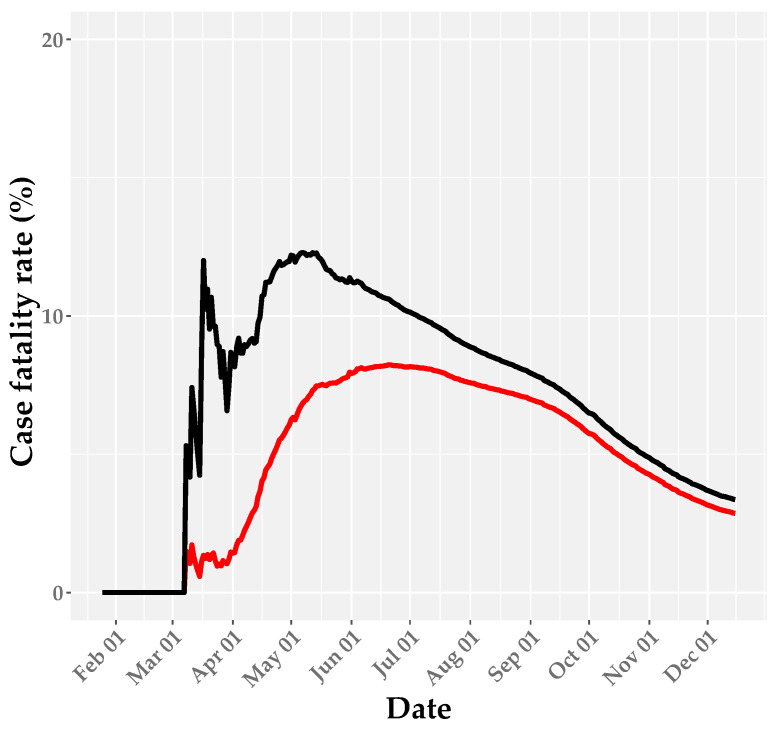
Crude case fatality rate (CFR; red line) and adjusted CFR estimates (black line) in Canada up to 15 December 2020. The shaded area around the black line illustrates the 95% credible interval (CrI) for the adjusted CFR.

**Figure 3:**
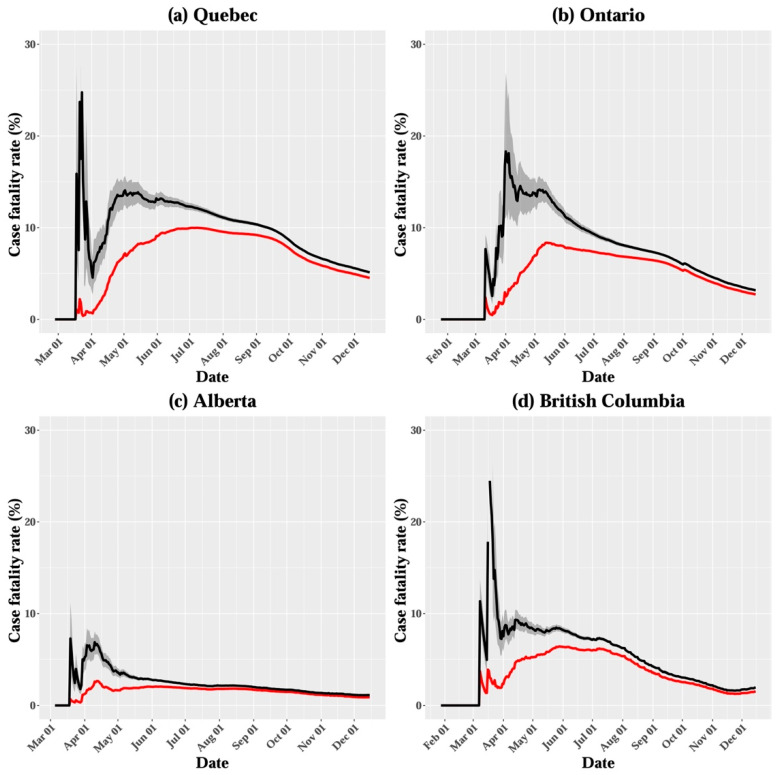
Crude case fatality rate (CFR; red line) and adjusted CFR estimates (black line) in (**a**) Quebec, (**b**) Ontario, (**c**) Alberta, and (**d**) British Columbia, up to 15 December 2020. The shaded area around the black line illustrates the 95% CrI for the adjusted CFR.

The crude CFR across all age groups in Canada was estimated to be 3.13% on 15 December 2020 (Table 2 and Figure 2). At the provincial level, the crude CFR was the highest in Quebec (4.5%), followed by Ontario (2.8%), British Columbia (1.5%), and Alberta (0.9%; Figure 3). When the survival interval was accounted for, the 15 December estimate of adjusted CFR in Canada was 3.36%, with a 95% credible interval (CrI) of 3.29–3.43% (Table 3 and Figure 2). The adjusted CFR tended to vary over the course of the epidemic, and it had considerably higher values than crude CFR in the early stage, likely owing to the delay in reporting confirmed cases, followed by a decreasing trend in all regions. The 15 December adjusted CFR varied considerably among provinces, ranging from 1.13% (95% CrI 1.09–1.17%) in Alberta to 5.13% (95% CrI 5.04–5.23%) in Quebec (Table 3). In addition to the adjusted CFR, the age distribution of cases and deaths differed at the provincial level, as indicated by the proportion of cases among elderly individuals (80 years and over), ranging from 3.5% in Alberta to 11.8% in Quebec (Table 4).

## 4. Discussion

The estimates suggest that the adjusted COVID-19 CFR in Canada is likely to be less than 4%; however, the estimates at the provincial level vary considerably. The results indicate that the most severely affected province in terms of confirmed cases and deaths has been Quebec, followed by Ontario, whereas Alberta and British Columbia have been less severely affected (Table 1). The latest estimates of the delay-adjusted CFR were highest in the two most populous provinces (i.e., Quebec and Ontario), where outbreaks were largely driven by infection transmission in LTC facilities. Specifically, it was reported that 709 LTC facilities (or 51% of all LTC facilities) in Ontario were affected, leading to 11,031 cases among residents, including 2800 deaths. There were 6467 reported cases among staff, including 8 deaths [28]. Similarly, in Quebec, COVID-19 cases were confirmed in 17,741 residents and 6324 staff members in 882 LTC facilities, leading to 6080 deaths among residents and 8 deaths among staff [28]. As a result, the proportion of elderly individuals among confirmed cases is relatively high in Quebec (11.8%) and Ontario (7.8%; Table 4). However, higher proportions of younger adults (aged 20−39) among confirmed cases were reported in Alberta and British Columbia. Thus, the results indicate variation in the age distribution of cases among provinces.

Although the estimates of delay-adjusted CFRs presented in the current study consider individuals of all ages in Canada, COVID-19 CFR estimates are known to be highly dependent on age and gender. The mortality distribution found in this study indicates that death from COVID-19 is age-dependent. Compared to younger groups, the crude CFR was considerably higher among elderly individuals, at an estimated 25.71%, and the proportion of deaths was also considerably higher (Table 2 and Table 4). Therefore, the adjusted CFR in Quebec and Ontario, where the proportion of elderly individuals among confirmed cases has been relatively high, tends to be higher than in other provinces.

In the current COVID-19 pandemic overall, it has been indicated that older age is a major risk factor for mortality. In particular, an age of over 70 years is associated with a markedly higher CFR [29,30,31]. In a prior study where the disease burden associated with COVID-19 was analyzed in 20 severely affected European countries, the United States, and Canada, it was shown that the crude CFR of COVID-19 is predominantly determined by patients aged 75 years or more [29]. It is also suggested that detailed information regarding the age distribution among confirmed COVID-19 cases needs to be taken into account, especially for countries with relatively low CFRs [29].

A large proportion of COVID-19-related deaths occurred through mid-June in Canada, of which approximately 80% involved residents of LTC facilities [32]. Our estimates of adjusted CFR are lower than prior estimates based on Canadian data—5.5% (95% CrI: 4.9−6.4%) from February to April 2020 [15]—partially because the estimates include data from recent outbreaks during the second wave, during which the average age of confirmed cases shifted towards younger age groups; therefore, there were fewer deaths that occurred compared to the first wave. Furthermore, recent data from British Columbia, Ontario, Alberta, Saskatchewan, Quebec, and Manitoba show an increase in COVID-19 infections among those aged 20−29 years, with more cases among women. Thus, further analysis of CFR stratified by age and gender would provide a more accurate measure of virulence and prediction of the burden of SARS-CoV-2.

A prior analysis attempted to adjust the delay in time between diagnosis and death, and estimated the CFR for 82 countries outside of China to be 4.24%, based on the number of cases in the 13 days prior to the assessment date [33]. Another study, using data from mainland China, adjusted for under-ascertainment and time delay, obtained a CFR of 1.38% for all ages, with a CFR of 6.4% for those older than 64 years [26]. Estimates of time-delay adjusted CFR in other countries were 5.33% (95% CrI: 5.00%−5.68%) in Japan [34], 6.1% (95% CrI: 5.4%−6.9%) in the United States [15], 9.1% (95% CrI: 8.9%−9.3%) in Peru [35], 10.2% (95% CrI: 9.0%−11.5%) in South Korea [16], 10.8% in Spain [36], 3.85% in China [37], 12.2% in Wuhan, China [38], and 17.8% in Lombardy, Italy [36]. These differences in CFR estimates could be associated with factors such as the timing of the estimates, age distribution within populations, different testing strategies, and the intensity of public health and social measures. Indeed, age differences in confirmed cases across Canadian provinces can also be partly attributed to different COVID-19 testing strategies. Quebec and Ontario primarily tested symptomatic individuals until March, after which they shifted their focus to healthcare workers and elderly patients in April to contain outbreaks in LTC facilities. Alberta, however, tested healthcare workers and LTC facility staff in early March, then expanded testing to all symptomatic individuals, and recently asymptomatic ones as well.

Some limitations of this study should be noted. It is likely that asymptomatic or very mild cases of COVID-19 were not reported, and thus not included in our analysis, possibly leading to an overestimated CFR; the transmission of SARS-CoV-2 from asymptomatic individuals (or individuals in the incubation period) has been well documented. In a prior study with a large-scale COVID-19 diagnostic testing of 9199 persons in Iceland, it was found that 43% of positive cases were asymptomatic [39]. Similarly, in another study, where a total of 215 pregnant women were tested, 29 of 33 (88%) who tested positive at admission were asymptomatic [40]. Nevertheless, asymptomatic individuals and individuals with mild symptoms are not routinely tested, leading to a potential overestimation of the CFR. In addition, the distinction between the number of tests performed and the number of individuals tested is not always clear in public data, which might reduce the impact of the bias because of the ascertainment of cases. Therefore, the preferential ascertainment of severe case bias in COVID-19, in addition to underreporting of cases, may have spuriously increased our estimated CFR, as has been shown in other studies [35,41]. Furthermore, a decreasing CFR might be reflective of increasing testing and a shift towards testing individuals with mild symptoms or asymptomatic cases [42].

Underreporting of causes of death may have potentially resulted in an underestimation of the CFR. For instance, the death tolls in Wuhan were revised on 17 April 2020, with 1300 fatalities added to the initial official count because of the inclusion of deaths that occurred at home or at institutions [43]. Similarly, it was estimated that approximately 4100 deaths of elderly people in LTC homes in Madrid, Spain were not counted in official reports because their reported symptoms were thought to be incompatible with COVID-19 [44]. In a prior study, to address the bias associated with the underreporting of the cause of death, all-cause mortality estimates were compared with those in previous years, and approximately 25% of comparative excess mortality was shown to be possibly attributed to COVID-19 in Germany and Portugal [45].

The reported CFR of COVID-19 tends to vary over the course of the epidemic. In general, the upward trend of the CFR during the early phase indicates increasing ascertainment bias. During a growing epidemic, the true CFR is underestimated early in the epidemic because the final clinical outcome of most reported cases is unknown. This pattern was observed in our results, as well as in prior studies of epidemics of respiratory pathogens, including SARS and H1N1 influenza [17,46]. The time-varying values of CFR could indicate that the risk of dying of COVID-19 (among detected cases) changes over time, but it could also imply compositional differences in the detected infections [47]. Nevertheless, an adjusted CFR is often relatively constant during a long period of imposed social distancing, providing useful insights for the prediction of fatalities and designing COVID-19 mitigation strategies.

## 5. Conclusions

The COVID-19 pandemic continues to impose a large death toll in Canada, disproportionately affecting the most populous provinces. When the delay between disease onset and death was accounted for, the estimates of the adjusted CFR (3.36% with 95% CrI: 3.29−3.43%) were higher than the crude CFR (3.13%), and varied substantially across provinces. Importantly, in Quebec, where the proportion of elderly cases was greater than 10%, the adjusted CFR was shown to be above 5%, and this estimate exceeded previous estimates for the global average CFR (2.9−3.1%) [48], as well as the average CFRs in Japan and the United States. Given the regional variations in CFR and its dependency on age, further studies with patient-level data on mortality and risk factors could provide a more detailed understanding of the factors shaping the risk of death related to COVID-19 in Canada.

## Figures and Tables

**Table 1:** Number of confirmed COVID-19 cases and deaths in Canada as of 15 December 2020, by province or territory. As of 15 December 2020, there had been 475,214 confirmed cases and 13,659 deaths attributed to COVID-19 in Canada, with confirmed cases in all provinces and territories; Quebec had reported the highest number of confirmed cases. Ten of Canada’s 13 provinces and territories had reported deaths, with Quebec and Ontario reporting the highest numbers.

Location	Total Cases	Deaths
Canada (total)	475,214	13,659
Quebec	167,276	7571
Ontario	144,396	3992
Alberta	83,327	744
British Columbia	43,463	668
Manitoba	21,535	508
Saskatchewan	12,432	98
Nova Scotia	1426	65
New Brunswick	559	8
Newfoundland and Labrador	359	4
Nunavut	258	0
Prince Edward Island	89	0
Yukon	59	1
Northwest Territories	22	0

**Table 2:** Distribution of COVID-19 cases by age group in Canada (as of 15 December 2020). Of the 475,214 cases reported in Canada, age information was available for 424,202 cases.

		Confirmed Cases, n (%)	Deaths, n (%)	Fatality Rate (%)
Total		424,202 (100.0)	13,280 (100.0)	3.13
Age Group	0–19	66,580 (15.7)	3 (0.0)	-
	20–29	78,782 (18.6)	15 (0.1)	0.02
	30–39	65,735 (15.5)	30 (0.2)	0.05
	40–49	61,657 (14.5)	86 (0.6)	0.14
	50–59	56,704 (13.4)	325 (2.4)	0.57
	60–69	36,072 (8.5)	977 (7.4)	2.66
	70–79	22,157 (5.2)	2456 (18.5)	11.08
	≥80	36,515 (8.6)	9388 (70.7)	25.71

**Table 3:** Time-delay adjusted case fatality rate (CFR) of COVID-19 in four provinces of Canada (Quebec, Ontario, Alberta, and British Columbia) and nationwide (as of 15 December 2020).

Area	Latest Estimate	Range of Median Estimates during the Study Period
Quebec	5.13% (95% CrI: 5.04–5.23%)	4.64–25.22%
Ontario	3.17% (95% CrI: 3.10–3.24%)	2.58–18.26%
Alberta	1.13% (95% CrI: 1.09–1.17%)	1.12–7.23%
British Columbia	1.97% (95% CrI: 1.89–2.05%)	1.61–30.38%
Canada (nationwide)	3.36% (95% CrI: 3.29–3.43%)	3.36–12.30%

**Table 4:** Distribution of COVID-19 cases and deaths in Canada by age group and province (as of 15 December 2020).

		Confirmed Cases (%)				Deaths (%)			
		Quebec	Ontario	Alberta	British Columbia	Quebec	Ontario	Alberta	British Columbia
Age group	0–19	17.0	12.6	19.4	12.9	0.0	0.0	0.0	0.0
	20–39	29.2	36.5	38.6	41.5	0.1	0.4	1.1	0.2
	40–59	28.0	28.7	27.8	28.0	2.2	4.0	3.0	3.8
	60–79	14.0	14.4	10.7	12.7	24.5	26.3	31.3	25.0
	≥80	11.8	7.8	3.5	4.9	73.2	69.4	64.6	71.0
	Total	100.0	100.0	100.0	100.0	100.0	100.0	100.0	100.0

## Data Availability

We obtained the daily number of confirmed cases and deaths associated with COVID-19 in Canada from publicly available sources, available at https://health-infobase.canada.ca/covid-19/epidemiological-summary-covid-19-cases.html (accessed on 10 January 2021).
